# Specificities of presentation of Crohn’s disease in childhood

**DOI:** 10.1590/S1679-45082017RC4070

**Published:** 2017-12-07

**Authors:** Bruna Karoline Pinheiro França Protásio, Camila Maria Pinheiro Machado Martins Barbosa, Clarice Blaj Neufeld, Leandro Dimasi Buck, Lygia de Souza Lima Laund, Mauro Sergio Toporovski, Thais Cristina Visoni

**Affiliations:** 1 Irmandade da Santa Casa de Misericórdia de São Paulo, São Paulo, SP, Brazil

**Keywords:** Colitis, Crohn disease, Prognosis, Child, Case reports, Colite, Doença de Crohn, Prognóstico, Criança, Relatos de casos

## Abstract

The incidence of inflammatory bowel disease in the pediatric population has increased in the last years. The most common form of inflammatory bowel disease is Crohn's disease and, according to its form and age of presentation, it is possible to predict the evolution of the disease.

## RESUMO

A incidência de doença inflamatória intestinal aumentou na população pediátrica nos últimos anos. A forma mais comum de doença inflamatória intestinal é a doença de Crohn e, conforme sua forma e a idade de apresentação é possível prever a evolução da doença.

## INTRODUCTION

Crohn's disease is an inflammatory bowel disease that causes transmural inflammation of the gastrointestinal tract.^(^ ^1^ ^)^ It is more severe in children than in adults, both in presentation and evolution, a fact that should alert the physician for an early diagnosis.^(^ ^2^ ^,^ ^3^ ^)^

Patients are classified as early-onset if the disease appears before 10 years of age. As to presentation, Crohn's disease is classified as stenotic, fistulizing, stenotic and fistulizing, or nonstenotic and nonfistulizing.^(^ ^4^ ^)^

The purpose of this case report is to describe the evolution of a pediatric patient when intestinal stenosis and fistula are present upon diagnosis of Crohn's disease.

## CASE REPORT

LFS, 10-year old female, mixed race. Cesarean birth, at 37 weeks, with no neonatal complications. Mother with adequate prenatal care, G1P1A0, nonconsanguineous parents, no comorbidities. No family history of autoimmune disease. Received a complete vaccination schedule. Proper neurodevelopment.

She presented abdominal pain, diarrhea with blood and mucus in stools, and weight loss, at 9 years of age. One month later, the patient underwent an esophagogastroduedonoscopy that revealed mild nonspecific chronic duodenitis; and a colonoscopy that showed deep ulcers interspersed with areas of normal mucosa, and some segments with coalescent lesions and narrowing of the intestinal lumen. A biopsy revealed moderate nonspecific chronic colitis. Due to the clinical, endoscopic and histological findings compatible with Crohn's disease, the patient was treated with prednisolone, at a dose of 2mg/kg/day, and mesalazine, at a dose of 40mg/kg/day.

The patient was referred to the pediatric gastroenterology outpatient clinic of the *Irmandade da Santa Casa de Misericórdia de São Paulo*, in São Paulo (SP). At the first visit, the patient had a body mass index (BMI) of 13kg/m^2^ (Z score <-3), and height of 129cm (Z score <-3), and she presented perianal abscess and fistulae. She scored 75 in the Pediatric Crohn's Disease Activity Index (PCDAI), which indicates severe disease activity. Other results: hemoglobin 9.3g/dL; hematocrit 27%; leukocytes 10,310/mm^3^; platelets 736,000/mm^3^; and C-reactive protein 94.4mg/dL. Infliximab was introduced, at a dose of 5mg/kg every 6 weeks; mesalamine had the dose adjusted to 60mg/kg/day. Metronidazole and ciprofloxacin were administered – the latter two drugs due to the perianal abscess. In the following 6 months, there was an improvement in the disease severity, with a reduction in the PCDAI score to light activity levels.

After one year, colonoscopy was repeated, showing the same results as the initial exam, the PCDAI score was 33.5 (moderate activity), and azathioprine was introduced at a dose of 2mg/kg/day. An abdominal magnetic resonance imaging (MRI) scan showed enteroenteric fistula and anorectal fistula. A remission induction of the disease activity was attempted with exclusive enteral nutrition, but the patient refused the treatment both orally and by nasogastric tube. Therefore, the treatment with infliximab, mesalamine and azathioprine was maintained.

After 2 years of diagnosis, due to persistent abdominal distension, early satiety, abdominal pain and loss of appetite, an abdominal MRI was performed, revealing distal ileum stenosis. She was submitted to laparotomy, which revealed multiple areas of fistulas and stenoses, which were resected. The patient evolved with clinical improvement during the following 6 months. However, after 3 months, she presented anemia, weight loss and poor appetite, and infliximab was increased to a dose of 10mg/kg every 4 weeks.

Four months later, she was admitted to the pediatric emergency room with intestinal subocclusion. She underwent surgical resection of the stenotic areas and fistulas, excising 10 centimeters of the ileum and transverse colon, besides the ileocecal valve. An ileostomy was performed.

The patient continued therapy with azathioprine and adalimumab, progressing to clinical improvement, normalization of hemoglobin (hemoglobin 14g/dL, C-reactive protein 0.2mg/dL), weight gain (BMI 21kg/m^2^), increase in height to 141.2cm, improvement of appetite and quality of life, PCDAI score 5 (no disease activity), and negative fecal calprotectin.

## DISCUSSION

Inflammatory bowel diseases are chronic, with peak incidence at ages 15 to 30 years. They are represented by three diseases: ulcerative colitis, Crohn's disease, and indeterminate colitis. The first two conditions have typical characteristics, and the third one has an unusual presentation that prevents a differentiation between Crohn's disease and ulcerative colitis, requiring a long-term follow-up to define the diagnosis.

In the United States, the incidence is approximately 4.5 per 100,000 inhabitants for Crohn's disease, and 2.4 per 100,000 inhabitants for ulcerative colitis. There are no estimated data in Brazil,^(^ ^3^ ^)^ with similar proportions in both sexes.^(^ ^5^ ^)^ A growing number of diagnoses has been observed in recent years, alerting pediatricians for the need to suspect and recognize an inflammatory bowel disease. The disease is more severe in children than in adults, greatly affecting their quality of life, not only by the manifestations of the disease but also by the effects of therapy.^(^ ^4^ ^)^

The etiology is a combination of genetic, environmental and immune factors.^(^ ^5^ ^)^ First-degree relatives are 20-fold more likely to develop the disease than the general population.^(^ ^6^ ^)^ In addition to this, other data reinforce the genetic inheritance of the disease, such as concordance for site and type of the disease in the same family.^(^ ^7^ ^)^

The genetic origin of Crohn's disease is based on changes in genes responsible for cell homeostasis (as IBD1), for regulating the immune response, and for the adaptive barrier.^(^ ^8^ ^,^ ^9^ ^)^ Environmental factors affecting the intestinal tract, such as type of childbirth, water supply and sewage conditions, feeding, antibiotic use, and smoking are triggers.^(^ ^10^ ^)^

Whereas ulcerative colitis only affects the mucosa of the colon, Crohn's disease transmurally affects any part of the gastrointestinal tract, and the most commonly involved areas are the terminal ileum and the right colon.^(^ ^3^ ^)^ In approximately 50% of cases, there is also involvement of the upper gastrointestinal tract. The onset of the disease results in deep ulcerations, with a non-continuous distribution, forming a typical colonoscopy appearance called “cobblestone”. The complications include stenoses and fistulas.^(^ ^2^ ^)^

The clinical manifestations of Crohn's disease include abdominal pain, diarrhea, hematochezia, lack of appetite, weight loss, fever, and perianal lesions, as well as extra-intestinal manifestations About half of patients have short stature and pubertal delay, which may precede intestinal symptoms.^(^ ^2^ ^)^

The diagnosis of Crohn's disease is established by a combination of clinical findings, laboratory tests, and endoscopic exams with biopsy.^(^ ^5^ ^)^ Another important aspect of diagnosis are imaging exams of the upper gastrointestinal tract, which, when affected, suggests the need for early surgery.^(^ ^10^ ^)^

Infliximab is a chimeric monoclonal antibody against tumor necrosis factor alpha (TNF-α), and it is effective to induce and maintain remission, but its long-term effect can be complicated by the loss of response, which occurs in 13% of patients. Adalimumab (human monoclonal antibody against TNF-α) may be an alternative in these cases.

When the patient already presents stenosis and perianal disease upon diagnosis, these medications have poor results. Recent studies showed that fistulizing and fibrostenotic phenotypes of Crohn's disease do not respond well to drug therapy and benefit from early surgery.^(^ ^10^ ^)^

## CONCLUSION

The patient was refractory to medical treatment, and only improved after two surgical interventions. Based on the scientific literature, we believe that the stenotic and fistulizing type of the disease, and the presence of perianal disease, were the factors that limited efficacy of infliximab. However, we cannot exclude loss of response to infliximab, due to antibodies against it, since we had no specific exams to confirm this fact.
